# Outcomes of importance to children and young adults with cerebral palsy, their parents and health professionals following lower limb orthopaedic surgery: A qualitative study to inform a Core Outcome Set

**DOI:** 10.1111/hex.13428

**Published:** 2022-01-27

**Authors:** Hajar Almoajil, Francine Toye, Helen Dawes, Jo Pierce, Andrew Meaney, Aziz Baklouti, Lara Poverini, Sally Hopewell, Tim Theologis

**Affiliations:** ^1^ Nuffield Department of Orthopaedics, Rheumatology and Musculoskeletal Sciences University of Oxford Oxford UK; ^2^ Department of Physical Therapy, College of Applied Medical Science Imam Abdulrahman Bin Faisal University Dammam Saudi Arabia; ^3^ Physiotherapy Research Unit, Nuffield Orthopaedic Centre Oxford University Hospitals NHS Foundation Trust Oxford UK; ^4^ Centre for Movement, Occupation and Rehabilitation Sciences, Faculty of Health and Life Science, Oxford Institute of Nursing, Midwifery and Allied Health Research Oxford Brookes University Oxford UK; ^5^ Oxford Health NHS Foundation Trust Oxford UK; ^6^ Faculty of Health and Life Sciences, Oxford Institute of Nursing, Midwifery and Allied Health Research Oxford Brookes University Oxford UK

**Keywords:** cerebral palsy, core outcome set, interview, surgery

## Abstract

**Introduction:**

Although several outcomes are commonly measured to assess the effect of surgery for young people with cerebral palsy (CP), these are selected mainly by health professionals and researchers. Including the perspectives of a broader range of stakeholders is an essential step towards determining important outcomes for assessment. This qualitative study involves the development of a core outcome set (COS) for lower limb orthopaedic surgery for ambulant children with CP.

**Objective:**

This study aimed to identify outcomes that matter to children and young people with CP, their parents and healthcare professionals following lower limb orthopaedic surgery.

**Methods:**

Semi‐structured interviews were conducted with 10 healthcare professionals, 10 children and young people with CP and 8 parents. Interview data were analysed by content analysis supported by the International Classification of Functioning, Disability and Health (ICF‐CY) supplemented by thematic analysis.

**Findings:**

Thirty‐one outcomes were identified in total, which were linked to eleven second‐level ICF‐CY categories. There were differences between stakeholder groups in preferences and expectations from surgical outcomes. Healthcare professionals and children with their parents identified 31 and 25 outcomes, respectively. Health outcomes valued by participants were lower limb alignment and symmetry, flexibility and muscle strength, mental health, fatigue, pain, function in life, mobility, participation, being independent, quality of life and adverse events. Compared to previous published trials, 10 new outcomes were revealed by this study.

**Conclusion:**

The researchers identified outcomes that are important to all stakeholders following lower limb orthopaedic surgery for ambulant CP. Including these outcomes in future studies would promote patient‐centred care for children and young adults with CP. Findings will be used to inform an international Delphi survey and develop a COS in this field.

**Patient and Public Contribution:**

This study was informed by an advisory group including a young adult with CP and a parent of a child with CP. This group engaged in the design of the study and the information material to support the interview (information sheet and interview topic guide).

## 1 INTRODUCTION

Cerebral palsy (CP) is the most common cause of childhood physical disability, affecting 2–3 individuals per 1000 live births globally.^1^, ^2^ Musculoskeletal deformities and resulting motor impairments are common and progressive during childhood, and lead to pathological and compensatory gait patterns.^3^ Many children with CP undergo lower limb orthopaedic surgery to address these musculoskeletal deformities and to improve or maintain mobility.^4^

The World Health Organization International Classification of Functioning, Disability and Health‐Children and Youth (ICF‐CY) provides a multifactorial assessment of health and health‐related status.^5^ However, assessment of outcome following lower limb surgery for ambulant children and young people is still based mainly on physical impairments, such as joint flexibility, alignment and gait improvement.^4^, ^6^ This does not account for the needs and expectations of children with CP and their parents, who are likely to focus more on activity and participation.^4^, ^7^ Consequently, there have been recommendations to include patient‐reported outcome measurements (PROMs) in clinical practice for children with CP.^7^

A recent systematic review identified substantial heterogeneity of outcomes reported in lower limb orthopaedic surgery research for children living with CP.^8^ This heterogeneity limits the possibility for meta‐analyses and the establishment of robust conclusions in this field. Core Outcome Sets (COS) have been proposed to address this problem by identifying a minimum set of outcomes in clinical practice and research.^9^, ^10^ A guiding principle in the development of a COS is that outcomes reflect the views and priorities of all key stakeholders, including patients and their representatives, alongside healthcare professionals, to maximize the relevance and impact of future research.^11^, ^12^

Few qualitative studies have previously been undertaken to develop priority‐based PROMs. A recent example is the Gait Outcomes Assessment List (GOAL),^11^ which reflects the patients' and parents' priorities in measuring outcomes after orthopaedic surgery. To our knowledge, no qualitative study has been undertaken to inform a COS in the field of lower limb orthopaedic surgery for CP. The role of qualitative research in the development of COS has been previously explored,^12^, ^13^ and has been advocated by groups such as the Core Outcome Measures in Effectiveness Trials initiative to ensure that the outcomes being considered for prioritisation are comprehensive.^9^

This study forms part of a COS development project, of which the protocol has been previously described and published.^14^ The first stage in the project involved identifying a ‘long‐list’ of potentially important outcomes. These were prioritized during the second stage, which included a Delphi survey and a final consensus meeting. In the study reported here, we explored the experiences, perceptions and priorities of stakeholders (children and young people with CP, their parents and healthcare professionals) who had been involved with lower limb orthopaedic surgery. The aim is to identify outcomes that have not been previously reported in the literature but matter to the relevant stakeholders. The findings informed the development of a COS in lower limb orthopaedic surgery for ambulant CP,^14^ and was conducted and reported using COnsolidated criteria for REported Qualitative research (COREQ).^15^

## 2 METHODS

### 2.1 Qualitative descriptive study

The researchers used a qualitative descriptive approach using content analysis of semi‐structured interviews with relevant stakeholders (health professionals, children with CP and their parents). A semi‐structured interview, guided by a list of essential topics, was chosen to facilitate open discussion and to avoid a closed question and answer format.^16^ The participants' experiences of relevant outcomes were explored.

Previous research with children with CP found that the concept of health outcomes was difficult for children to grasp quickly;^17^ therefore, a child‐friendly technique using Talking Mats (https://www.talkingmats.com/) was used with the children (8–15 years) to enhance participation and interest in answering the questions.

This study was approved by the Research Ethics Committee (19/SC/0357) and received R&D approval from the relevant NHS Trust.

### 2.2 Study sample and sample size

A purposive approach to sampling was used to recruit stakeholders with a range of demographic characteristics such as children's age, Gross Motor Function Classification System (GMFCS) level,^18^ surgery type and age at surgery, health professional's background and years of experience. The sampling strategy aimed to capture a range of views concerning the outcomes of orthopaedic surgery treatment. The sample included children with CP and their parents, health professionals specializing in CP lower limb orthopaedic interventions and health researchers with an interest in CP or children with disability. Table 1 presents the sample criteria. The sample was drawn from a leading hospital in the UK specializing in orthopaedic surgery for children with CP.

**Table 1:** Participants' inclusion criteria

**Health professionals**
Heath professional with at least 2 years' experience in CP treatment ○ Orthopaedic surgeons ○ Paediatrics rehabilitation teams (e.g., physiotherapist, occupational therapist) ○ Nurses Researchers in the field of CP
**Children and representative (parent, carer)**
Diagnosed with cerebral palsy Age from 8–18 years old at the time of the surgery Ambulant, that is, within levels I–III of Gross Motor Function Classification System Have received or are being considered for orthopaedic lower limb surgery Parent or carer of a child or young person fulfilling the above criteria

There are no agreed criteria for determining sample size in qualitative research.^19^ Participants were included in the study until data saturation was reached. Data saturation was defined in this study as the point at which three consecutive interviews generated no additional data.^20^, ^21^

### 2.3 Recruitment

Health professionals and researchers based at the tertiary hospital where recruitment was conducted were invited to participate in the study if they fulfilled the criteria (Table 1). An email invitation was sent to potential participants by the research team. Participants were offered the opportunity to have their interview in private, either at the hospital or at the university department. Informed consent was obtained before the interview and confidentiality was maintained throughout the study.

Children and their parents, who attended the paediatric orthopaedic service at the hospital between October 2019 and June 2020 and fulfilled the criteria (Table 1), were approached by their direct care team during the child's consultation appointment. The invitation included an information sheet indicating that participants could withdraw from the study at any time. Children were given the choice to be interviewed with or without a parent present. Written consent was obtained from all participants (children and parents) on the day of the interview. Participants' demographic information was collected during the interview to ensure that the sample fulfilled the purposive sampling criteria.

### 2.4 Interviews

A semi‐structured interview guide was developed with a range of stakeholders, including specialists in CP orthopaedic surgery, a social scientist, researchers, children and young adults with CP and parents of a child with CP to ensure that all questions were open, relevant and sensitively worded. The interview guide included a series of open‐ended questions based on a topic guide to ensure that key areas were covered (File S1).

Interviews initially focused on the participants' current day‐to‐day life experiences and surgical interventions related to lower limbs. Participants were asked to reflect on their priorities after surgery, how these priorities changed following their surgical intervention and how their priorities might differ compared to other stakeholders' groups.

To facilitate the discussion and communication during the children's interviews, the ‘Talking Mat’ was used.^22^ This is an interactive resource using picture symbols to facilitate communication and provide a structured framework for open questions. Previous research has examined the feasibility of using Talking Mats with a range of conditions, including CP, aphasia and intellectual and learning disabilities.^22^, ^23^, ^24^ The researcher (H. A.) collaborated with a play specialist to create a set of picture symbols, which represented different health outcomes.

**Figure 1:**
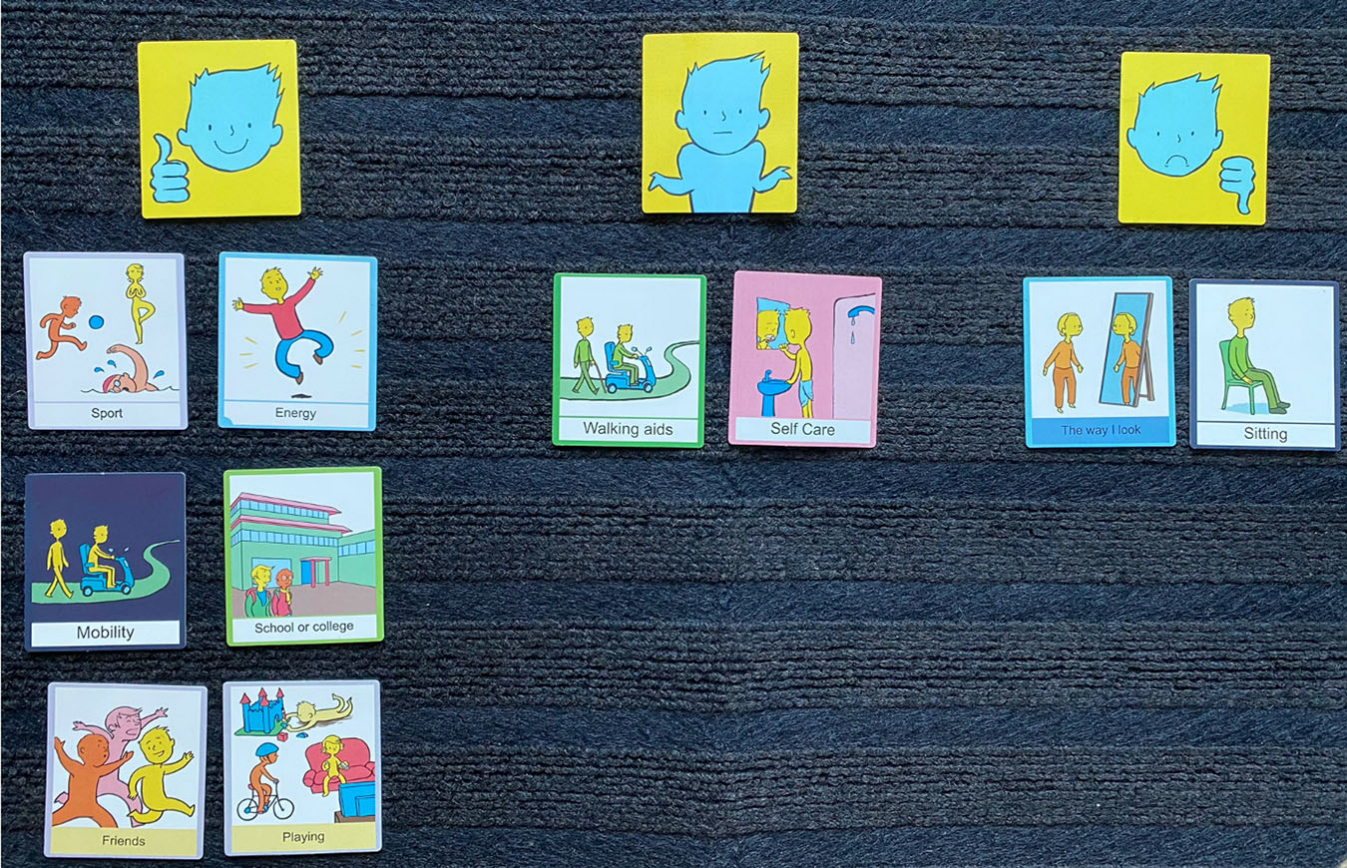
Results of Talking Mat showing a child's priorities postsurgery

The Talking Mat technique involved placing a mat in front of the child. This was divided into three columns, each headed by a response category to indicate their general preference and to express their views about each topic^22^, ^23^: ‘important’ (thumb up and smiling face), ‘not sure’ (a child shrugging his shoulder) and ‘not important’ (thumb down and sad face). The children were shown the picture of the specific activity and then asked how they felt about each item: They indicated their choice by placing the picture under the relevant response category. The resulting Talking Mats image was photographed as a record of the participant's view on each topic (Figure 1), and interviews were tape‐recorded.

All participants were interviewed by the same researcher (H. A.). Most interviews were undertaken at the hospital or the participant's home. However, due to the coronavirus pandemic (COVID‐19), after March 2020, five interviews with children and parents were conducted online using Microsoft Teams.

Each interview was audio‐recorded and transcribed verbatim. Participants were identified by their participant ID code to maintain confidentiality. Critical and reflective feedback on early interview transcripts was given by an experienced qualitative researcher (F. T.) to facilitate the development of ideas during ongoing interviews.

### 2.5 Data analysis

A qualitative content analysis was performed^25^ using a deductive approach, with the ICF‐CY as a framework to categorize the data.^5^ The interviews were read through to obtain an overview of the data and to identify *meaningful concepts* from the interviews. In this context, ‘Meaningful concepts’ refer to those that reflect the essence of what participants are saying. These meaningful concepts were linked to the most relevant ICF‐CY categories following a set of formal rules and procedures as determined by Cieza et al.^26^ In addition, inductive thematic analysis^27^ was performed to develop new categories from the meaningful concepts that did not fit the chosen framework.

### 2.6 Linking to the ICF‐CY framework

According to established linking rules,^26^ each line of text was coded according to its meaningful concepts and was linked to an ICF‐CY category. Some data could be linked to more than one ICF category. For example, for the statement ‘for the ambulatory kids, they can have discomfort because of their spasticity or because of joint problems. So that would always be my prime goal’, the meaningful concepts would be ‘muscle spasticity’ and ‘joint problem’, and the ICF categories linked to them would be ‘b735 muscle tone function’ and ‘d710 mobility of joint function’. If the meaningful concept was not explicitly named in the ICF‐CY category, the ‘nc; not covered’ category was applied. If there was insufficient information to decide about which ICF category should be linked, the concept was assigned as ‘nd; not defined’ and this included concepts referring to the quality of life (nd‐qol).

### 2.7 Thematic analysis

Meaningful concepts that could not be linked to ICF‐CY categories (nc, nd or nd‐qol) were pooled together and analysed thematically.^27^ Thematic analysis is a process where meaningful concepts are compared and organized into categories of ‘themes’, which share a common essence.

### 2.8 Accuracy and rigour of the analysis

To ensure consistency of linking results, identification of meaningful concepts and linking of ICF‐CY categories, the linking was conducted independently by two qualitative researchers trained in using the ICF‐CY classification system. For data that did not fit the ICF‐CY framework, two researchers placed meaningful concepts into categories. Differences arising in interpretation between the researchers were resolved through discussion.

## 3 FINDINGS

### 3.1 Participants and interviews

A total of 20 interviews were conducted (10 health professionals, 8 children and parent dyads, 2 young adults with CP individually). Participants' demographic data are shown in Tables 2 and 3. All professionals were specialists in treating children with CP and lower limb deformities, with their experience ranging from 3 to over 20 years. The age range of the children interviewed was 10–17 years. The severity of the condition, based on the GMFCS level, varied: Level I (*n* = 1), Level II (*n* = 5) and Level III (*n* = 4). Talking mats were used in three interviews. An example of a talking mat is shown in Figure 1. Interviews lasted from 25 to 53 min.

**Table 2:** Characteristics of the sample (health professionals)

Participant	Gender	Health professional		Length of the interview (min)
		Years of experience	Professions	
HP 1	F	11–20	Allied health or nurse	31
HP 2	F	≤10	Allied health or nurse	44
HP 3	F	>20	Allied health or nurse	37
HP 4	F	11–20	Researcher	25
HP 5	F	>20	Surgeon	34
HP 6	M	≤10	Allied health or nurse	39
HP 7	M	>20	Surgeon	39
HP 8	F	11–20	Researcher	45
HP 9	F	≤10	Researcher	53
HP10	M	>20	Surgeon	32

Abbreviations: F, female; HP, health professional; M, male.

**Table 3:** Characteristics of the sample (children and young adults)

Children and young people							Parents/carers		Length of the interview (min)
Code	Gender	Age (year)	GMFCS	Previous surgery	Time from surgery	Talking mat	Code	Relation to the child	
CH01	F	14	II	SEMLS	Presurgery	Not used	P01	Father	29
CH02	F	14	III	SEMLS	3.6 years	Not used	P02	Mother	33
CH03	F	17	I	Soft tissue^a^	Presurgery	Not used	P03	Mother	45
CH04	M	16	III	SEMLS	Presurgery	Not used	P04	Mother	45
CH05	M	10	II	SEMLS	Presurgery	Used	P05	Mother	46
CH06	M	15	II	SEMLS	3 years	Not used	P06	Mother	56
CH07	F	17	III	SEMLS	8 years	Not used	P07	Mother	50
CH08	M	11	II	SEMLS	5 years	Used	P08	Father	43
CH09	M	16	II	SEMLS	2.6 years	Not used	–	–	33
CH10	M	15	II	SEMLS	1 year	Used	–	–	53

Abbreviations: CH, children; F, female; M, male; P, parent; SEMLS, single event multilevel surgery.

^a^ Hamstrings, gastrocnemius lengthening.

### 3.2 Outcomes identified

Thirty‐one outcomes were generated from the data, and these were organized into five categories. Two categories were linked to the ICF‐CY framework: (1) body function and structure and (2) activity and participation. Three additional categories were generated from a body of data that did not fit the ICF‐CY framework: (3) independence, (4) quality of life (QoL) and (5) adverse events. A list of the identified outcomes for each group is presented in Table 4.

**Table 4:** Desired outcomes for each stakeholder group and those identified from the scoping review^28^

Outcomes identified	Healthcare professionals	Children and families	Scoping review
Body function and structure			
Mental health	•	•	
Gait pathology	•		•
Joint mobility	•	•	•
Lower limb alignment	•	•	•
Muscle strength	•	•	•
Muscle tightness	•	•	•
Pain level	•	•	•
Fatigue/energy drive movement	•	•	•
Activity and participation			
Activities			
Mobility level	•	•	•
Changing body position	•		•
Using stair	•	•	•
Standing in all forms	•	•	•
Reduce fall incidence	•	•	
Balance	•	•	
Daily life function	•	•	
Self‐care	•	•	•
Walking	•	•	•
Participation			
School activity engagement	•	•	•
Social satisfaction	•	•	•
Sport/hobby	•	•	•
Within home	•	•	
Other			
Independent	•	•	
Quality of life			
Emotional well‐being	•	•	
Aesthetics and self‐esteem	•	•	
Parent's burden	•	•	
Adverse events			
Recurrent surgery	•		•
Infection	•	•	•
Fracture	•		•
Neurovascular symptoms	•		•
Healing process	•	•	•
Pressure sores	•		

### 3.3 Body function and structures

#### 3.3.1 Alignment and symmetry

Some participants described improved lower limb alignment and symmetry as an important component of the surgery outcome. These improvements could have an impact on activities (e.g., better mobility) or complications, such as the development of scoliosis due to poor symmetry.

The important results were that…, my knees were much straighter, in a straightened position, …, They're important to me in a massive way, because it's so helpful in daily life. CH09

In contrast, some health professionals were doubtful that correction of joint alignment would lead to functional improvement, but rather, they thought that it was important because parents and children felt that symmetry was integral to a positive body image.

If one leg's turning in or something. It won't necessarily be because it impairs them functionally, but they are very conscious that it makes them look different. HP04

#### 3.3.2 Flexibility and muscle strength

Participants felt that increased joint mobility and muscle strength were important outcomes of the surgery. Similarly, they felt it was important to manage muscle spasticity postoperatively. Participants emphasized that these musculoskeletal impairments would have an impact on other symptoms, such as pain.

Hopefully then by doing that [surgery], the muscles will be able to strengthen, and she'll be able to walk longer distances without needing her chair. P01

#### 3.3.3 Mental health

Participants described how orthopaedic surgery can negatively affect a child's mental health. There was a sense that it was important to maintain the child's mental health postoperatively, and some felt that there was a lack of mental health support after surgery.

I have seen multilevel surgery induce mental health problems on children with probably an underlying problem and it came up on the surface as a result of the stressful experience on the orthopaedic treatment. HP10

#### 3.3.4 Fatigue

Health professionals and children described reducing ‘fatigue’ or increasing available ‘energy’ as important outcomes. They noted the negative consequences of fatigue on daily and social activities and emotional functioning.

If I use my energy, I can't walk or I can't breathe or I can't… energy means a lot to me, it's in my body. If I don't have energy, I can't run around, I can't walk. I can't go out and chase my friends around or tickle them or play with them. I need energy so I can do stuff. CH08

One surgeon described the complexity of defining fatigue and distinguished between *general* fatigue and *muscle* fatigue.

Muscle fatigue is something that orthopaedic surgery is supposed to improve by reducing the energy cost of walking. So, in theory, when you improve the mechanics of the legs you reduce the energy costs of walking that should reduce fatigue. But general fatigue, the feeling of fatigue, is a much more complex phenomenon. It is not just dependent on energy cost. There are other parameters. Some of them being psychological. HP10

#### 3.3.5 Pain

Participants described reduction of pain as an important outcome of surgery, as pain could have a negative impact on daily activities, social and family life. However, there was a sense that pain was often insufficiently controlled or measured.

Pain affects sleep. That impacts on parents. That impacts on relationships at home. That impacts on the child's involvement with the rehab. It impacts on their participation in school, participation in life, basic life. HP02

My legs were burning every day and they couldn't do anything about that. So, I had to go through some burning pain. CH09

### 3.4 Activity and participation

#### 3.4.1 Function in life

Participants described the children's ability to function optimally as an important outcome postoperatively. Although their level of function would not be expected to be ‘normal’, it should be at a level that would allow them participation in activities they deem important.

Whether they've got sort of a range of 30 degrees or 40 degrees. If they're able to actually function with that, that's what's key. HP03

#### 3.4.2 Mobility

Participants highlighted the importance of the children maintaining mobility level postoperatively. Mobility was defined as the ability to sit, stand, transfer and move around. Although mobility was considered as an important outcome, some felt that time constraints in clinical practice were a barrier to assessing a child's mobility level postsurgery.

My main goal is to know that I can step off a thing and on a thing, and even if not stairs, even if it was a little tiny sledge, I know I'll be able to try and do it. That's one of my short‐term goals. CH04

Maintaining body balance during mobility and reducing the incidence of falls were considered important postoperatively. Both children and parents described a fear of falling, and of being alone in the event of a fall. Participants also described how falls could have a negative impact on the child's confidence, leading to reduced independence.

It's almost like I've got agoraphobia almost, not that I can't leave my house, just I'm scared of walking around an open space where I feel like I might fall over. CH06

Participants considered walking ability as an important outcome following lower limb orthopaedic surgery. Walking was described as a multidimensional outcome that could include several different aspects, for example, the ability to walk further, faster or better, with or without the use of an assistive device. While walking ability was considered important as an immediate outcome of surgery, health professionals raised the issue of maintaining walking improvement into adulthood.

Hoping to maintain walking ability long into the future… What I don't want to do is do a big operation on somebody, improve their walking for two or three years, and for that then to decrease again when they're adults. HP05

Some participants highlighted the importance of being able to walk with better leg alignment. As such, measurement of the quality of the gait would be important. Others highlighted the importance of improving endurance and being able to walk further, either with or without assistive devices. Health professionals emphasized the need to measure gait parameters objectively, and some noted the available outcome measures to evaluate gait abnormality, such as the three‐dimensional gait analysis. Although these measures would provide valuable clinical information, they felt that this information might be less meaningful to children and parents.

[the] key focus is to get mechanically their walking as good as possible, which mostly means making their pattern as close to normal as possible. HP04

Health professionals emphasized that they would not expect surgery to reduce the need for an assistive walking device and highlighted situations where children's and parents' expectations of walking capacity postoperatively were not aligned. They described differences in these views between children and their parents, mainly related to the use of assistive devices. In contrast, some participants felt that using an assistive advice would increase independence.

As an example of this finding, one young adult and her mother described striving towards walking independently as:

I haven't had someone say, ‘Well, you won't be able to walk independently’, but I've also had the physiotherapist say, ‘Oh. Well, probably, our goal for you is to be walking with sticks’. And I said, ‘Well, I don't really want to walk with sticks, so it's not a goal I want to go for’. Because, if I'm working so hard, and then that is as good as I get. CH07

Your goal would be to walk unaided. But, of course, that has to be tempered with a big dose of realism. And I think for [child's name] to go from where she was to being able to walk completely unaided would've been unrealistic. P07

#### 3.4.3 Participation

Participation and involvement in everyday activities and social life were highlighted as a key outcome. Several aspects of participation were discussed, for example, within the home, school or community environment. Opportunities to be involved in recreation and leisure activities were considered key aspects of social inclusion. In addition, the fundamental role of participation in sport was emphasized because of its impact on physical health.

[I] would probably measure participation. So, whether it's school, if they're going to things with the family, sort of social schooling and family. HP08

It's to help [child's name] sort of be able to do more, to be more active, to be more sporty, to enjoy her sport and dance, to enable her to do more things. P01

### 3.5 Independence

Being independent in daily life activities, self‐care and walking were considered as important postoperative outcomes. Some felt that it was important to achieve as much independence as possible during childhood and noted the efforts of parents to help children achieve independence.

Independence is a big thing because before my third year, a lot of people, especially my family had to rely on helping me every day. That would involve helping me walk, helping me change my clothes because I couldn't, it was very difficult at that time because I couldn't stand up properly. CH09

Moreover, health professionals and children described how difficulties in the perioperative period, particularly during rehabilitation, would challenge personal autonomy and subsequently have an impact on QoL.

I think it's come up in the sense of autonomy. It's not independence. An autonomy comes up a lot, which is being in control. So, deciding you're going to do something. I think it's probably that's how I've come across that a lot all the time. HP08

### 3.6 Quality of life

QoL was described as an important outcome by participants. However, it was highlighted that making judgements about somebody else's QoL is subjective. Health professionals and parents also linked QoL with components of the ICF‐CY framework, for example, improvement in the child's physical health and social life, including the independent mobility and the ability to retain a social network.

Participants described the child's emotional well‐being as a main component of QoL and an important outcome. Children could experience stress, fear, anxiety and depression after surgery. Some described exhaustion following major surgery as an important factor affecting the QoL, which could have a negative impact on the child's and family's emotional well‐being.

I would measure a kind of one mood construct. So just about how they're feeling with regards to anxiety, depression. HP08

Participants described how feelings and emotions could affect the QoL. Some children compared their body and their activities to those of their siblings and friends. As such, aesthetics was reported as an important postoperative outcome, which in turn increased the child's self‐esteem.

Aesthetics is important. That's about self‐esteem, and that is hugely important. HP05

Low self‐esteem was also experienced as an outcome of surgery. A 17‐year‐old young adult and his mother spoke about lost motivation and low self‐esteem:

The quality of life is very important because there are a lot of things in life that I'm trying to accomplish. A couple of years ago I wanted to become a scientist. I wanted to become the one that used to cure cancer to help people like me. But now, because in my school, they used to give me science classes, but because of the way I am now, I have to come in later which means I'm missing those classes, which means my passion for science went down and because I'm basically stuck in my room for maybe hours a day. CH04

His self‐esteem went down about that because he used to say to me, ‘What scientist is in a wheelchair?’ And I'd say to him that your wheelchair is your legs, so you are no different to anybody else. P04

### 3.7 Adverse events

Adverse events following surgery were described as important and integral to the process of balancing the benefit and risk of treatment and guiding informed consent. Health professionals highlighted the need to systematically report surgical complications, both clinically and in research.

Participants discussed complications that should be measured postoperatively, for example, infection, fracture, wound problems, nerve and blood vessel damage and pressure sores.

I think that is an extremely important aspect of the management. It is very much part of the consenting process before surgery. It is something that should always get up on the scales when weighting the pros and cons of surgery. HP10

She went through a bit of a tough time afterwards because we were constantly coming back to try and sort out the infections of the wounds. P02

## 4 DISCUSSION

This study used a qualitative approach to identify 31 surgical outcomes that are important to children and young people with CP, their parents and the healthcare professionals looking after them. These were categorized under five core areas: body function and structure, activity and participation, independence, QoL and adverse events.

Participants viewed outcomes as interrelated, with some of them affecting higher‐level outcomes. For example, participants described the importance of improving joint flexibility and muscle strength to be more active, as this in turn would improve QoL. The inter‐relationship of outcomes has previously been identified and described for children with neurodisability.^17^ This highlights the need to consider and identify the degree to which higher‐level outcomes reflect primary clinical outcomes. These primary clinical outcomes are the stepping‐stones that lead to the achievement of higher‐level outcomes and longer‐term changes. Our findings indicate the need for a comprehensive assessment approach as highlighted by the ICF‐CY, to illustrate the interaction between outcomes and their impact.

Our findings included ten additional outcomes, beyond those identified in a recent scoping literature review (Table 4).^28^ In particular, participants considered surgery in relation to the ‘whole’ person and attached special significance to outcomes relating to mental health, emotional well‐being, independence in activities of daily life, participation and life impacts, rather than outcomes aligned to body function and structure, which have dominated the literature. This suggests that the current outcome reporting in clinical studies fails to report outcomes that are important and relevant to children and their caregivers. In contrast, the outcome priorities identified in this study, such as achieving independence, resonate with findings from previous qualitative work in children with neurodisability.^17^, ^29^

A tendency to report clinical outcomes and not life impact is a common feature of published studies. For example, although the impact of surgery on the child's QoL was considered important in this study, it was not reported in any of the 44 studies published in a systematic review.^28^ There may be several reasons why QoL is not routinely reported: First, the complexity of the concept, which encompasses social, emotional, cognitive, physical and functional well‐being; second, uncertainty about what should be measured to determine QoL.^30^ It may also be that improvement in other domains is a key factor towards achieving a better QoL.

Although our findings indicate that there is overlap between outcomes valued by different stakeholders, it appears that different stakeholders place emphasis on different outcomes and that healthcare professionals emphasize outcomes according to their own field of expertize. For example, surgeons focus on outcomes related to body structure: nurses focus on adverse events postsurgery: therapists focus on activity and participation. Similarly, parents might be concerned about their child's future independence and integration into the community, whereas children and young adults focus on areas of immediate relevance and their current ability. This variation in focus has been shown in previous studies.^29^, ^31^, ^32^

The range of identified outcomes indicates the importance of having a multidisciplinary approach and engaging children and their parents in decision‐making. It also highlights the importance of clear open communication in the decision‐making process for surgical interventions and the establishment of treatment goals.^33^ Our findings support the use of a combination of clinical and PROMs, such as the GOAL questionnaire,^34^ in the evaluation of surgical interventions and clinical trials. Similarly, we support the use of qualitative approaches to complement quantitative research findings, and to provide detail on surgical impact from the individuals' perspective. Incorporating stakeholder's views about valued outcomes is vital to facilitate a patient‐centred approach in healthcare. This is in line with the aims of generating a list of outcomes that are relevant to all stakeholders and that includes the voice of children and their parents. This will help to ensure that outcomes of lower limb surgical interventions for CP are not restricted to treating gait pathology and joint range of motion, but that they include life impact outcomes such as function, participation and QoL dimensions of health.

## 5 STRENGTHS AND LIMITATIONS

This qualitative study preceded the consensus phase of developing a COS to be used in the field of lower limb orthopaedic surgery.^14^ The study identified additional outcomes that had not been reported by a recent literature review on CP surgical trials.^12^, ^35^ The coding and analysis were undertaken by a minimum of two experienced researchers. Our methods were established a priori in a study protocol that had been subjected to a robust peer‐review process.^14^

A potential limitation of this interview study is that the participants were recruited from a single orthopaedic hospital. However, this hospital is a tertiary referral centre covering a wide geographic area of South England. There may be outcomes that are relevant to other national and international stakeholders that have not been identified. We limited the interviews to UK‐only participants for pragmatic reasons. A future consensus study (Delphi survey) that will form part of the COS development will be available internationally.

Qualitative research does not report statistical findings and does not aim to represent a particular population: Rather, it aims to generate ideas from a purposive sample that will enhance our understanding of a particular experience. Sociodemographic data were not collected for this study. The strength of our sample was that it was drawn from a specialist orthopaedic centre that serves a wide geographical area: Our primary aim was to develop a list of important outcomes from a purposive sample of stakeholders with experience, or professional interest, in CP. Future studies to explore the impact of different demographic variables, including ethnicity, education and income, on preferences and experiences would be useful.

Most of the children and parents were interviewed together, and this may have had an impact on the responses of both parent and child, which may have been different if they had been interviewed alone. However, the team considered that the most ethical approach was to give the children the choice to undertake an interview with or without a parent. This decision was in line with guidance following ethical review.

## 6 CONCLUSION

This study identified 32 outcomes that are important to all stakeholders involved with lower limb orthopaedic surgery in CP. Some of these have not been previously identified or used in trials within this study field. It is important that future studies in this field should report on outcomes that are considered important by children, young people and their parents.

## CONFLICT OF INTERESTS

The authors declare that there are no conflict of interests.

## AUTHOR CONTRIBUTIONS

Tim Theologis is responsible for the management of the study and is the principal investigator for the study. Helen Dawes, Francine Toye, Sally Hopewell and Tim Theologis supervized the field research. Hajar Almoajil, Francine Toye, Helen Dawes and Tim Theologis contributed to the design of the study. Hajar Almoajil, Andrew Meaney and Tim Theologis contributed to data collection. Hajar Almoajil, Francine Toye, Jo Pierce, Aziz Baklouti and Lara Poverini contributed to data analysis. Hajar Almoajil drafted the manuscript with significant inputs from all coauthors. All authors reviewed and approved the final version of the manuscript.

## ETHICS STATEMENT

This study was approved by the Ethics committee (19/SC/0357) and R&D approval was obtained from the relevant NHS Trust.

## Supporting information

Supplementary information.

## Data Availability

The data that support the findings of this study are available on request from the corresponding author. The data are not publicly available due to privacy or ethical restrictions.
