# Supplementary material for: Outcomes of importance to children and young adults with cerebral palsy, their parents and health professionals following lower limb orthopaedic surgery: A qualitative study to inform a Core Outcome Set
Source: Health Expect. 2022 Jan 27;25(3):925–35. doi: 10.1111/hex.13428 (PMC9122398; doi:10.1111/hex.13428)
Supplement: Supplementary file 1 — Supplementary information. [file HEX-25--s001.pdf]

Core Outcome Set (Interview Topic Guide)
Healthcare Professionals

Introduction

- Go over the purpose of the study with participant.
- Check they are still willing to take part.
- Check they are happy for interview to be audio recorded.
- Answer any other queries.
- Ask them to sign the consent form.

Interview Themes/Questions

General Question

1. What are some of your experiences with children and young people (CYP) with cerebral palsy throughout the time you have cared for them? These can apply to any age group for any musculoskeletal treatment for their lower limb/to improve their gait.

Probe
- Based on experience, what are some considerations you typically have with these patients? And or their carers

Assume participants will provide some experiences they have with this population

General experiences on outcomes

2. Overall, what lower-limb orthopaedic aspects of CYP's health matter most to you when working with this population, specifically for their lower-limb deformity?

Assume participants will provide some unique outcomes or considerations for this group

3. What are concerns or experiences that CYP/carers typically share with you post-surgery?

Probe
- This may include topics that are related to the CYP and/or family/carer, on physical health, daily routine or quality of life, etc

Assume participants will share some concerns that raised by patients and carers

Specific Questions on outcomes

4. Are there any differences between what you and the CYP/carers deem important?

Follow up questions:
- What might be the causes of any of these differences?

Expect discussions on outcomes that they considered important, where not relevant or important to the patients/carer

5. Which considerations do you have that you think other professionals/carer/patients over- emphasize, or under-emphasize?

Assume a discussion of general outcomes, in terms of what should be appreciated more or less

6. What are the five outcomes you value most when it comes to lower-limb surgery of CYP with cerebral palsy, and should definitely be measured in the research area? and why?

Expect valued outcomes and discussion

7. Moving to the systematic review results, outcomes that are reported in the literature include: [example of outcomes]. Do you have any opinions on any of these lists in general?

Assume discussion on some outcomes from the systematic review results

Closing questions

8. To sum up, after discussing and listing various perspectives and outcomes, could you provide me with two or three outcomes that you believe essential to consider?

Expect most valued outcomes list

9. Is there anything else you would like to add?

Closing up interview

- Thank you for participating.

Core Outcome Set (Interview Topic Guide)
Parent/carer

Introduction

- Go over the purpose of the study with participant.
- Check they are still willing to take part.
- Check they are happy for interview to be audio recorded.
- Answer any other queries.
- Ask them to sign the consent form.

Interview Themes/Questions

General Question

1. I understand you have a child with cerebral palsy, please could you describe some experiences about how your life has been affected? Are there any experiences, which have significantly changed the way you live as an individual, or in terms of family, friends?

Follow up question
- What has the treatment of your child's leg problems been like?

Assume participants will provide some experiences they have as parent/carer of a child with cerebral palsy with this condition and treatment, to stimulate conversation on the topic in general.

General experiences on outcomes

2. Overall in terms of your child's health, what matters most to you when considering orthopaedic surgery for his/her leg problem(s)?

Assume participants will provide some of main priorities

3. Is there anything that you have been thinking about related to your child's leg surgery that you feel hasn't been addressed yet in your discussions with the treating team?

Probe
- Are there any outcomes/issues/concerns you expected treatment to address but have not been considered?

Assume either no input, or some example of outcomes/concerns not been addressed.

Specific Questions on outcomes

4. When you discussed the results of your treatment with the team that treated your child, were there any issues where you didn't agree with them? Were there aspects of the treatment that you considered important and they did not, or the other way round?

Probe:
- Which aspects do you think have been over-emphasised?
- Was there anything that needs to be more emphasised?

Follow up questions:
- What do you think might be the cause of any of these differences?

Expect outcomes that they deem important, that healthcare professionals may not consider.

5. When you think of the treatment for your child's leg problems through surgery, what are the most important results you would wish them to achieve?

Follow up question
- Why do you think these results are important?
- Has what you consider important changed over time?

Expect list of valued outcomes and subsequent discussion

6. Outcomes that are reported from other young people with your child's condition include: [example of outcomes], what are your thoughts on those?

Follow up question
- Have you discussed any of these with your doctor?

Assume discussion on some outcomes from the systematic review results

Closing questions

7. To sum up, after discussing and listing various aspects of results and outcomes, could you provide me with two or three main issues that you think essential to consider?

Expect most valued outcomes list

8. Is there anything else you would like to add?

Assume either no input for this question, or further discussion

Closing up interview

- Thank you for participating.
- Providing participant £20 voucher and sign the voucher receipt.

Core Outcome Set (Interview Topic Guide)
Children and Young People (16 -1 8 years)

Introduction

- Make sure the accessibility for the child mobility is available [e.g., accessible entrance and adjusted seat].
- Go over the purpose of the study with participant.
- Check they are still willing to take part.
- Check they are happy for interview to be audio recorded.
- Prompt for and answer any other queries.
- Ask them to fill in the consent form.

Interview Themes/Questions

General Question

1. I understand you have cerebral palsy, please could you describe some experiences about how your life has been affected? Are there any experiences, which have significantly changed the way you live as an individual, or in terms of family, friends?

Follow up question
- What has the treatment of your leg problems been like?

Assume participants will provide some experiences they have with this condition and treatment, to stimulate conversation on the topic in general.

General experiences on outcomes

2. As you progress towards your adulthood, are there any more considerations, concerns or different experiences that arise for you?

Follow up question
For outcomes that may emerge:
- Which of these have been the most important to you?

Assume participants will provide some highlighted experiences and share some outcomes that may have emerged.

3. What are the main pieces of advice you would share with a friend, who has a similar condition, particularly about going through leg surgery?

Potential Probe
- We're interested in hearing about your expectations from the treatment, the care you need, and any issues related to your health or well-being?

Assume participants will share major experiences and considerations.

Specific Questions on outcomes

4. When you discussed the results of your treatment with the team that treated you, were there any issues where you didn't agree with them? Were there aspects of your treatment that you considered important and they did not, or the other way round?

Probe:
- Which aspects do you think have been over-emphasised?
- Was there anything that needs to be more emphasised?

Follow up questions:
- What do you think might be the cause of any of these differences?

Expect outcomes that they deem important, that healthcare professionals may not consider.

5. When you think of the treatment for your leg problems through surgery, what are the most important results you would wish to achieve?

Follow up question
- Why do you think these results are important?
- Has what you consider important changed over time?

Expect list of valued outcomes and subsequent discussion

6. Outcomes that are reported from other young people with your condition include: [example of outcomes], what are your thoughts on those?

Follow up question
- Have you discussed any of these with your doctor?

Assume discussion on some outcomes from the systematic review results

Closing questions

7. To sum up, after discussing and listing various aspects of results and outcomes, could you provide me with two or three main issues that you think essential to consider?

Expect most valued outcomes list

8. Is there anything else you would like to add?

Assume either no input for this question, or further discussion

Closing up interview

- Thank you for participating.
- Providing participant £20 voucher and sign the voucher receipt.

Core Outcome Set (Interview Topic Guide)
Children and Young People (8 -15 years)

Introduction

- Make sure the accessibility for the child mobility is available [e.g., accessible entrance and adjusted seat].
- Go over the purpose of the study with participant.
- Check they are still willing to take part.
- Check they are happy for the interview to be audio recorded.
- Prompt for and answer any other queries.
- Ask the parent to fill in the consent form and the child complete the age appropriate assent form.

The questions asked will be based on the talking mat tools **(talking mat example, version 01, date 25 /07/2019)**. As such these questions are possible examples of the type of questions that will be asked. Therefore, these questions will not be used precisely as they are and may be modified depending on the outcome of the activity, patient age and understanding.

Interview Themes/Questions

General Question

1. Introduce to talking mat

2. How do you feel about [different cards]? Within a scale of “important, unsure, not important” pictures, table 1 & 2.

Follow up question
- is there anything else that is not here, but is important to you?

Assume participants will share their experiences and discuss outcomes that may have emerged.

Different question about it:
How do you think about your mobility around home, school,.. etc? How do you feel about ...

Table 1: Questions with different activities

Using technologies	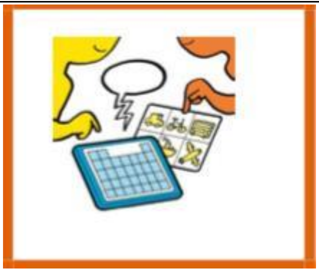
Socialising with friend/family	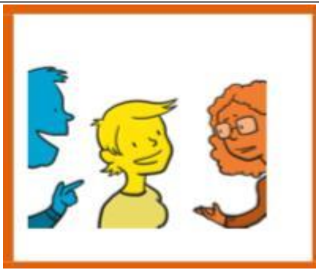
Participation with peers	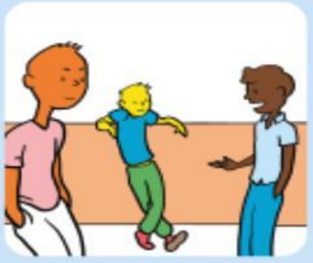
Bicycle (sport)	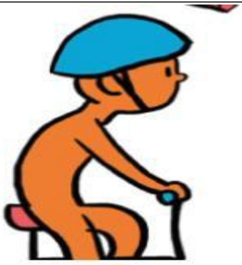

Table 2: Scale

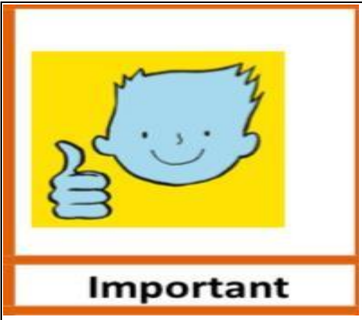	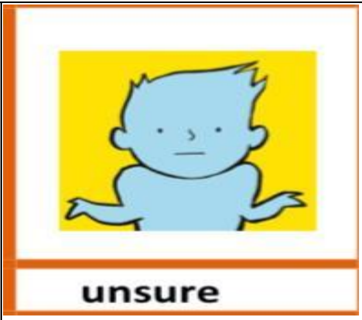	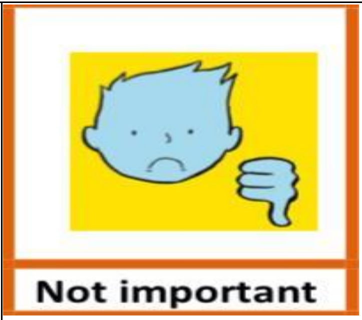
Assume different activities will be considered as important.	Assume different activities will be considered as unsure.	Assume different activities will be considered as not important to participants.

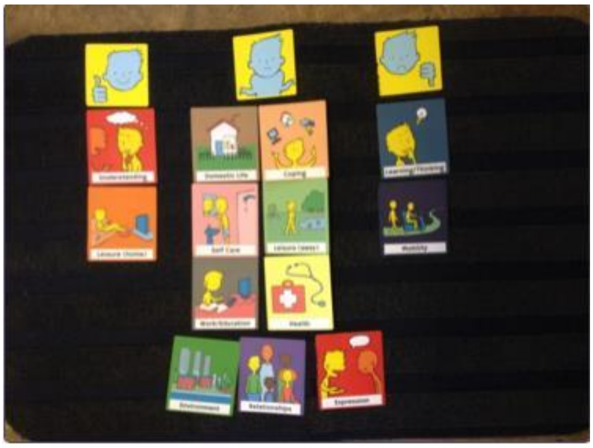

An example of a mat for the expected results will be on the same pattern

General experiences on outcomes

3. Choose which symbols you like the best [from important group]? From the completed activity above (Q2).

Follow up question
- Why do you like these activities?

Assume participants will provide some activities that are important to them.

4. How do you think about [different activities shown in cards]? These items were identified from someone else in your age or a family member, and/or doctors.

Potential Probe
- Of all cards you find important, are there any that your family/ doctors also find important?
- Can you tell me why?

Expect outcomes that they deem not important to them, that healthcare professional, family members may consider as important.

Specific Questions on outcomes

5. Are there any activities that you used to do but no longer doing because of your leg problem?

6. Did the surgery fix these problems and you able to do it now? Or Do you expect your future leg surgery will fix these problems and you will become able to do these activities?

Assume discussion over their experience with the condition and related surgery

7. What are the most important results [from cards] you would wish to achieve?

Follow up question
- Why do you think these results are important?

Expect list of valued outcomes and subsequent discussion

8. How do you feel about how long things took to be achieved after your leg surgery?

Assume participants will be share some experience about goals achievement.

Closing questions

9. To sum up, could you pick up two or three main things that you feel it is important to achieve after you had leg surgery?

Expect most valued outcomes list

10. Is there anything else you would like to talk about? Closing up interview

Closing up interview

- Thank you for participating.
- Providing participant £20 voucher and sign the voucher receipt.
